# Prosthetic Rehabilitation on Edentulous Patients with Microstomia: About Three Cases

**DOI:** 10.1155/2019/9578083

**Published:** 2019-04-21

**Authors:** Oumaima Tayari, Jamila Jaouadi, Safa Jemli, Hela Haloui, Ali Ben Rahma

**Affiliations:** Complete Removable Prosthodontic Department, Research Laboratory LR12ES11, Faculty of Dental Medicine, University of Monastir, Monastir, Tunisia

## Abstract

Limited oral opening is an acquired or congenital abnormal condition that compromises patient esthetics, nutrition, and quality of life. In addition, it may hinder conventional prosthetic procedures of edentulous patients, make it challenging, and present difficulties at all its stages. This clinical report presents different clinical treatment options suitable to be chosen by the prosthodontic carer in the case of reduced oral aperture.

## 1. Introduction

The reduction of oral opening (microstomia) is a change seen in a minority of patients [1]. It can occur due to electrical, thermal, or chemical burns, maxillofacial trauma, surgical treatment for orofacial neoplasms and reconstruction of lip defects (cleft lips), head and neck radiotherapy, microinvasion of the muscles of mastication or temporomandibular joint (TMJ) dysfunction syndrome, genetic disorders such as partial duplication of chromosome 6q, Hallopeau–Siemens-type recessive dystrophic epidermolysis bullosa, Freeman–Sheldon (whistling face) syndrome, Burton skeletal dysplasia, and diseases such as Plummer–Vinson syndrome or scleroderma and connective tissue diseases such as systemic sclerosis or other systemic disease [2–5].

Due to the restricted mouth opening, patients often face difficulty with eating and sometimes speech. In addition, it is difficult for them to insert and remove the prosthesis [5, 6].

In the other hand, this condition presents a challenge for the clinician through all the prosthetic treatment stages. It may be impossible to make impressions and fabricate dentures using conventional methods, so a modified denture approach, depending on the cause and severity of microstomia, must be employed [7–9].

Literature is abundant with various techniques of prosthodontic management of limited oral opening, and this paper aims at presenting different prosthodontic options for managements of edentulous patients with microstomia.

## 2. Case Report 1

**Figure 1:**
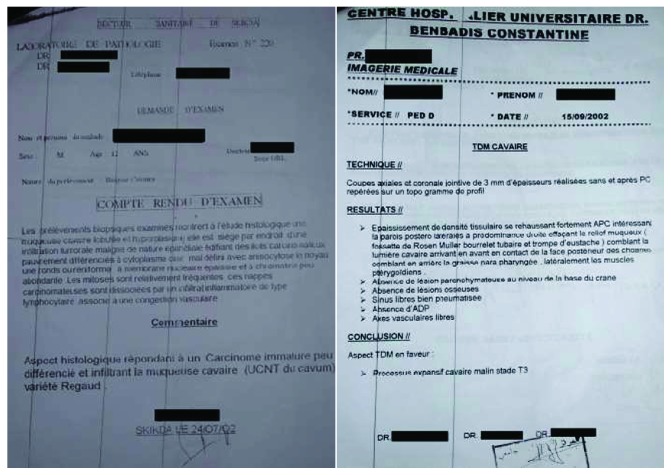
Histological and CT scan results.

A 26-year-old male was referred to the Department of Prosthodontics, Faculty of Dental Medicine, University of Monastir, Tunisia, for prosthetic rehabilitation. He was suffering from microstomia resulting of radiotherapy about 12 years ago following cavum carcinoma classified T3N0M0 (Figure 1). The patient had no experience about removable complete prosthesis rehabilitation.

**Figure 2:**
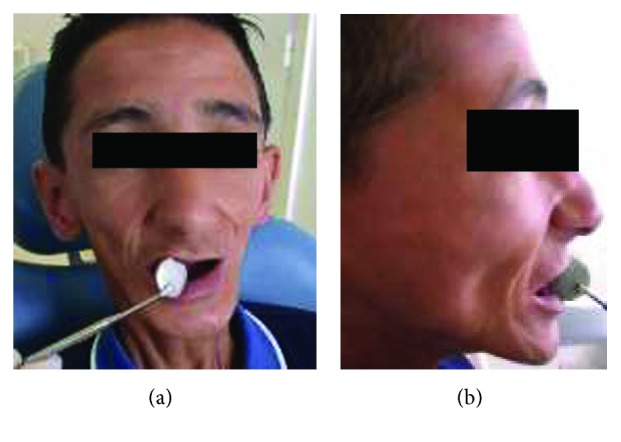
Preoperative views of the patient with severe limitation of oral aperture. (a) Frontal view; (b) side view.

Oral examination revealed a limited maximal mouth opening (MMO) measuring about 21 mm, with tight and inflexible labial tissues (Figures 2(a) and 2(b)).

He presented complete edentulous maxillary and mandibular resorbed ridges.

**Figure 3:**
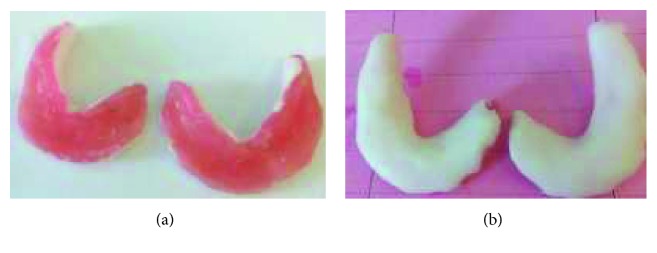
Sectional special mandibular tray. (a) Under side view (soft wax); (b) upper side view (autopolymerizing resin).

Restricted oral access made it difficult to make primary impression using classic stock impression trays. Hence, the choice was made to fabricate a mandibular sectional impression tray for each half of the arch by manually molding soft wax to the ridge, intraorally (Figure 3(a)). Then, the sectional special trays were reinforced by the autopolymerizing acrylic resin extraorally (Figure 3(b)). They were inserted into the patient's mouth in two separated pieces: left and right.

For the maxillary arch, the impression tray was made in one piece as the same manner as the mandibular one.

The preliminary impressions were made with irreversible hydrocolloid impression material (alginate).

**Figure 4:**
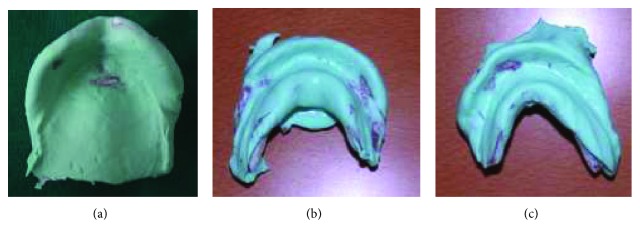
Primary impressions. (a) maxillary primary impression; (b) Right sectional mandibular impression; (c) left sectional mandibular impression.

The maxillary impression was made on one step (Figure 4(a)).

The two mandibular hemi-impressions were made separately (Figures 4(b) and 4(c)). Recording the anterior ridge, in addition to the corresponding side, by each hemi-impression is an indispensable condition to validate it.

First, the right mandibular hemi-impression was poured with type II dental plaster to obtain the right sectional primary cast. After it was set, and thanks to the central common area of the two hemi-impressions, the left hemi-impression could be positioned on the right sectional cast and poured. It was held with finger pressure until plaster was set ensuring not to displace the first sectional cast seated in the impression. So, we got an entire primary cast.

**Figure 5:**
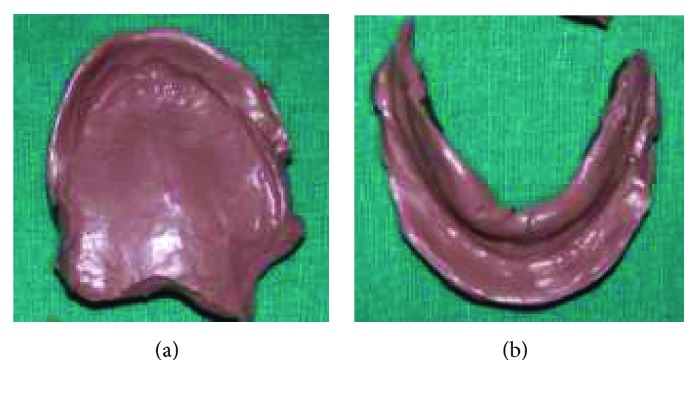
Final impressions. (a) Maxillary impression; (b) mandibular impression.

For the secondary impression, single custom trays were fabricated for each arch using autopolymerizing acrylic resin. Once placed intraorally, it was carefully positioned onto denture-bearing areas and molded to appropriate contour using functional and manual manipulation. Care was taken during border molding to correctly record the peripheral extension, as the vestibular depth and width were shallow (Figures 5(a) and 5(b)). Final impressions were poured to get final casts.

**Figure 6:**
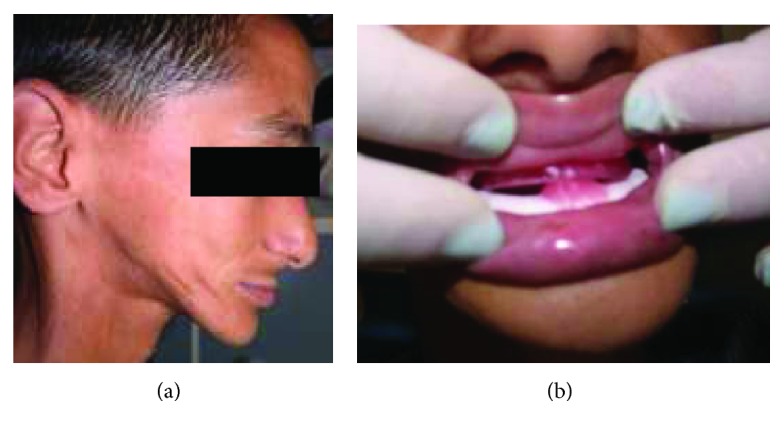
The maxillomandibular relationship record (OVD, CR). (a) Lateral view with established vertical dimension of occlusion; (b) intraoral view.

Mandibular movements become restricted because of facial skin fibrosis, muscular atrophy, and temporomandibular joint damage, which make recording the maxillomandibular relationship problematic. By making the mandibular record base, as possible, shorter and thinner and with patient cooperation, jaw relation record was obtained with the use of occlusion rims oriented to the established vertical dimension of occlusion, the anatomic occlusal plane, and the patient's centric relation (Figure 6(a)). The mandibular wax occlusal rim thus obtained was very short because of the reduction of the vertical prosthetic space which complicated the teeth arrangement (Figure 6(b)).

**Figure 7:**
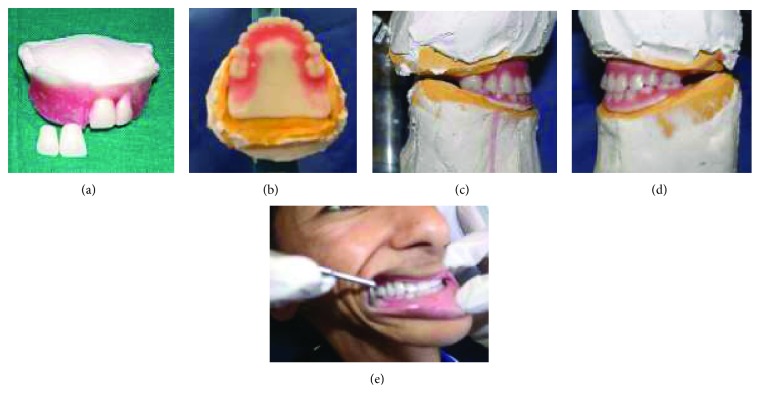
Teeth arrangement and try-in. (a) Teeth size reshaping; (b) teeth formula reduction (1 premolar and 1 molar); (c) right view; (d) left view; (e) try-in.

Because of a lack of teeth in appropriate sizes and shapes, conventional artificial teeth were reshaped to be managed in the available transversal and vertical space (Figures 7(a)–7(d)).

During the try-in stage, the stability and occlusion were verified and the esthetic aspect was accomplished (Figure 7(e)).

**Figure 8:**
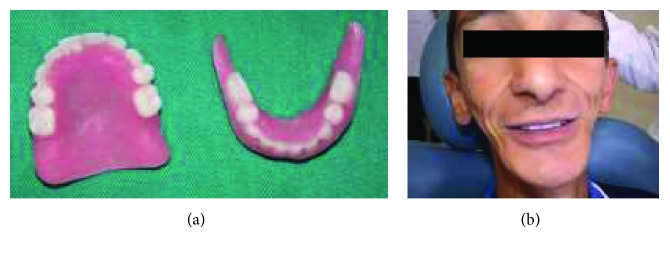
(a) Final dentures; (b) frontal view of the patient after insertion.

After teeth arrangement, complete dentures were fabricated with the usual laboratory procedures using heat polymerized polymethyl methacrylate resin. After bench cooling, the dentures were finished and polished (Figure 8(a)).

At the insertion appointment, the balanced occlusion was adjusted (Figure 8(b)). Oral hygiene instructions and prosthesis insertion and removal were imparted. Routine follow-up appointments were scheduled after a week. The patient was satisfied with the esthetics and functional ability of the dentures.

## 3. Case Report 2

**Figure 9:**
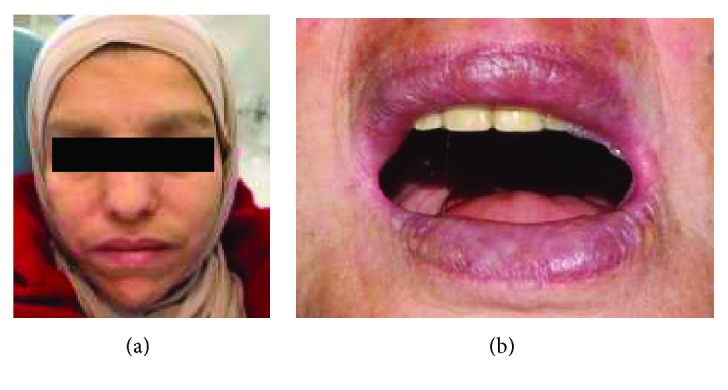
Preoperative view of the patient. (a) Frontal view; (b) maximal mouth opening.

A 47-year-old edentulous female consulted at the prosthodontic department of the Faculty of Dental Medicine of Monastir, Tunisia. She had extremely unuseful maxillary complete denture (Figure 9(a)).

Her lips were stretched, and she had a limited oral opening with a diameter and circumference of approximately 27 mm and 98 mm, respectively, resulting from cervical radiotherapy (Figure 9(b)). Her alveolar crest was sufficiently high, but her buccal and labial mucosas were thin and the saliva quantity and flowability were inadequate.

The treatment plan consisted of fabricating complete dentures with proper extension of the flanges.

With effort, preliminary impressions for both arches were obtained with flexible impression trays: a putty silicon impression material was inserted, and once placed intraorally, it was carefully molded by finger pressure covering the entire important anatomic regions using functional and manual manipulation.

**Figure 10:**
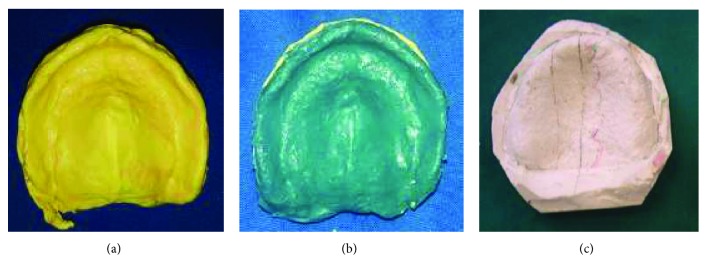
Maxillary preliminary impression. (a) First step of primary impression with putty silicon material; (b) second step of primary impression with light silicone material; (c) primary maxillary cast.

Due to flexibility, the silicone tray could be easily removed (Figure 10(a)).

A light silicone material was injected on the first impression, then, the light body wash impression was made by reinserting the silicone tray in the oral cavity (Figure 10(b)).

The custom trays for the final impressions were fabricated with autopolimerized acrylic resin on each primary stone cast (Figure 10(c)).

For the mandibular impression, the oral opening was sufficient for the custom tray to be inserted on the patient's mouth.

**Figure 11:**
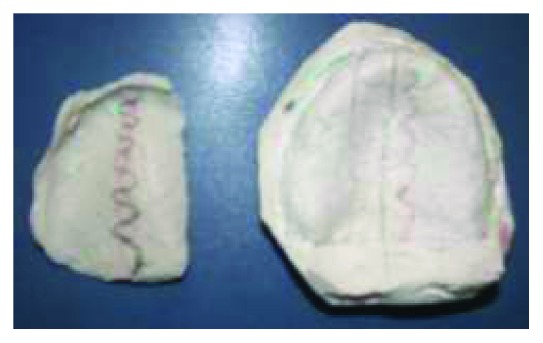
Left impression tray segment.

The maxillary custom tray was cut into two unequal sections, so that the labial frenum is recorded accurately in the impression. The two segments of the tray are joined using nick and notches (Figure 11). This facilitated the reassembling of the impressions outside the mouth. The insertion and movement to interlock the tray segments should be rehearsed in the mouth prior to the impression procedure.

The maxillary impression tray was inserted into the patient's mouth in two separate pieces, left and right. The fit of the borders was evaluated. Then, the peripheral borders of the trays were trimmed so that they were 2 mm shorter than the depth of the vestibule to allow space for the border-molding material. This latter was separately done for each half.

**Figure 12:**
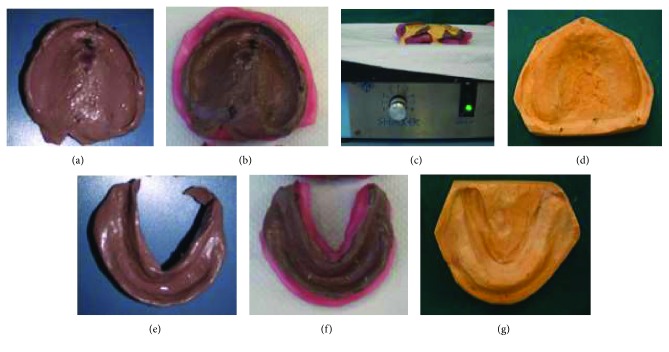
Final impression treatments. (a) Joined sectional maxillary impression; (b) maxillary impression coffrage; (c) maxillary impression pouring; (d) maxillary final cast; (e) mandibular final impression; (f) mandibular impression coffrage; (g) mandibular final cast.

Final maxillary impressions were made by using polysulfide material alternately for the first and second halves of the sectional trays. After the impression paste set, the right and left tray segments were removed separately. The acrylic resin halves were carefully joined extraorally, and minor discrepancies at the seam were filled with soft impression wax (Figure 12(a)).

Final impressions were boxed and gently poured to get final casts (vibrator) (Figures 12(b)–12(g)).

On final casts, record bases were fabricated and occlusion rims were prepared.

**Figure 13:**
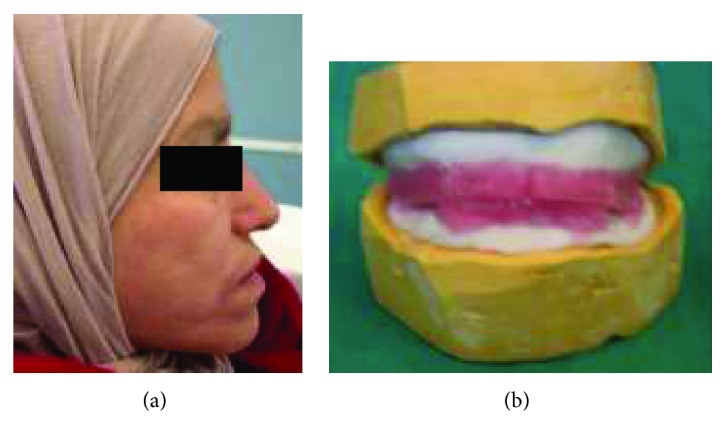
Occlusal record. (a) Lateral view with established vertical dimension of occlusion; (b) extraoral view.

After making a centric relation record, transferring the record from the patient to a semiadjustable articulator was done (Figures 13(a) and 13(b)).

**Figure 14:**
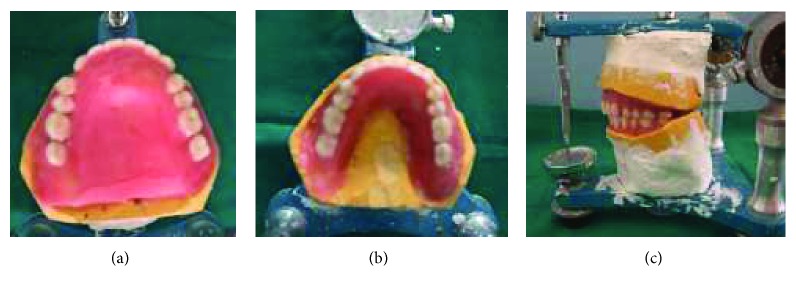
Teeth arrangement. (a) Maxillary view; (b) mandibular view; (c) lateral view.

Because of the lack of the prosthetic vertical space between the arches, the record bases were removed from the casts and replaced with thin slices of wax that supported the teeth arrangement (Figures 14(a)–14(c)).

The try-in was evaluated to verify jaw relations and teeth arrangement. After patient acceptance, final festooning and flasking of the dentures were completed.

**Figure 15:**
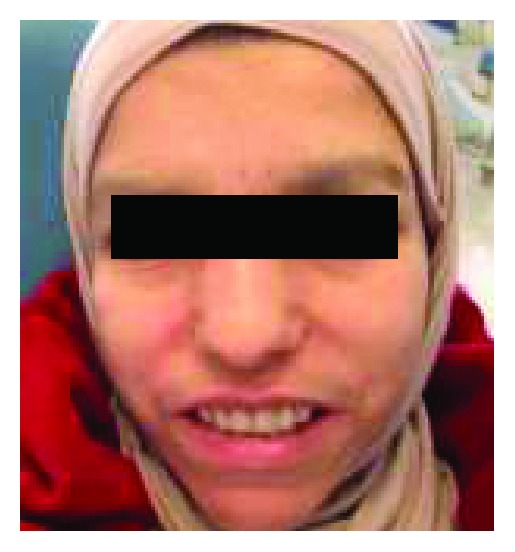
The postoperative view of the patient rehabilitated with a set of functional and esthetic complete dentures.

The final dentures were manufactured using similar designs to that of the record bases. The prostheses were deflasked, finished, and polished. The patient was provided with instructions for cleaning, inserting, and removing the prostheses (Figure 15). She was recalled for follow-up visits to check for the maintenance and her adaptability with the new dentures. Initially, the patient felt difficulty in the insertion and removal of denture, but progressively, she became used to.

## 4. Case Report 3

**Figure 16:**
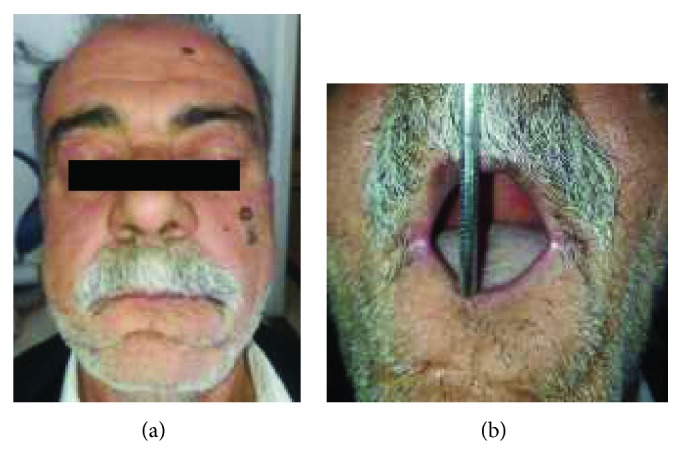
Preoperative view of the patient. (a) Frontal view; (b) maximal mouth opening.

A 56-year-old edentulous patient presented to our Department of Prosthodontics. He reported esthetic and functional difficulties. Extraoral examination revealed a small vertical oral aperture measuring 30 mm due to the presence of scars on the coetaneous side of the lower lip after surgical resection of a squamous cell carcinoma complicated by an active mentalis muscle (Figures 16(a) and 16(b)).

Maxillary and mandibular ridges were found to be large and well-formed. And as the patient could insert the mandibular denture into his mouth by rotating it 90 degrees, a conventional design was used.

With some amount of difficulty, conventional procedures for fabricating a set of complete denture were followed until the stage of occlusion record.

**Figure 17:**
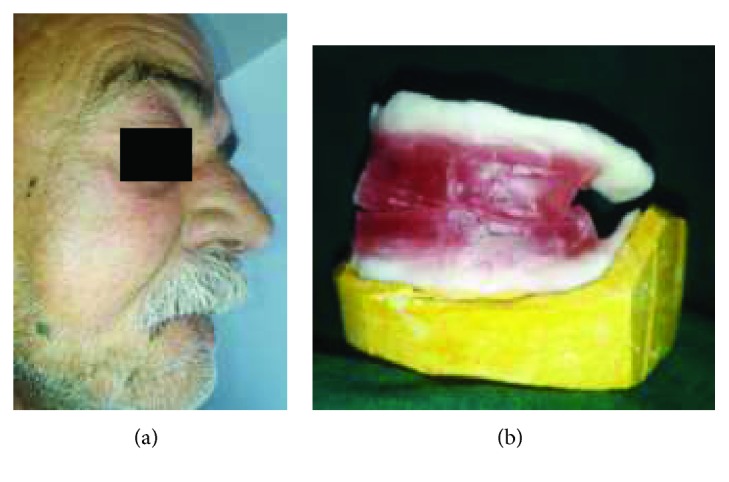
Occlusal record. (a) Lateral view with established vertical dimension of occlusion; (b) sagittal view of the occlusal rim in centric relation.

After centric relation recording, we found that the lower lip was interposed under the upper occlusal rim although there is a sagittal coincidence of the vestibular side of the two occlusal rims (Figures 17(a) and 17(b)).

**Figure 18:**
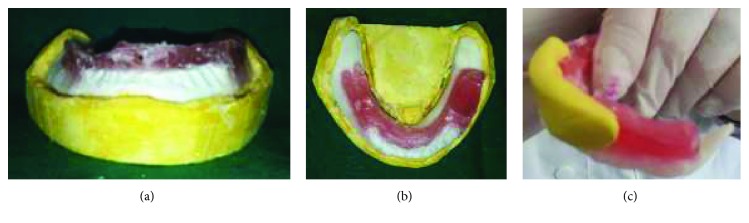
Mandibular occlusal rim modifications. (a) Frontal view of the modificated record base; (b) upper view of the modificated record base; (c) putty silicone in place.

So, the buccal side of the mandibular rim was arranged by removing the vestibular wax, trimming the record base, and perforating the occlusal rim (Figures 18(a) and 18(b)) to make a space to the putty silicone material (Figure 18(c)).

**Figure 19:**
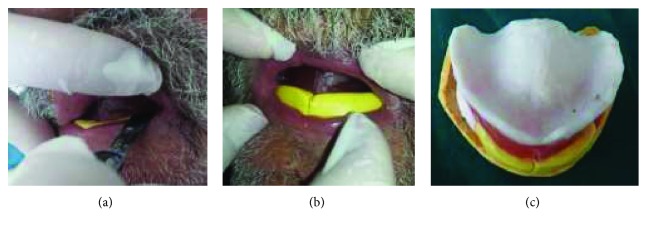
Second occlusal record. (a) Occlusal plan setting; (b) midline setting; (c) extraoral view.

The silicone was molded intraorally until having sufficient esthetic aspect and lower labial support. New jaw relation was recorded. The occlusal plane and the midlines were established (Figures 19(a) and 19(b)). The mandibular occlusal rim was placed more buccally than the maxillary rim (Figure 19(c)).

**Figure 20:**
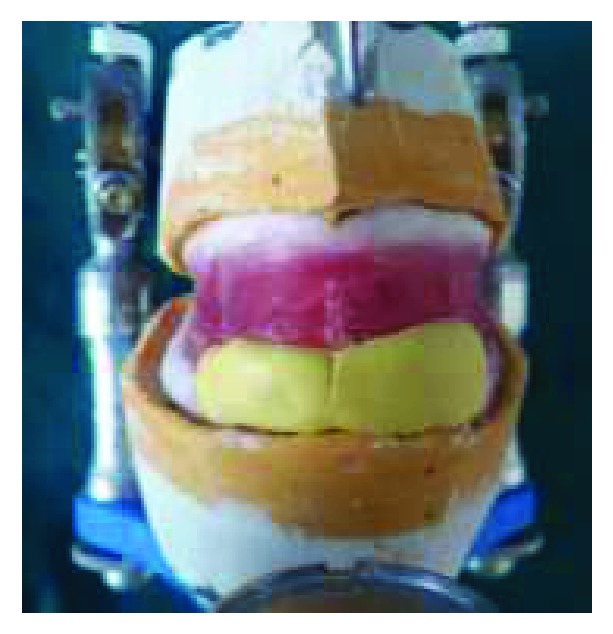
Definitive casts mounted on semiadjustable articulator.

Definitive casts were mounted on a semiadjustable articulator by using the maxillomandibular relationship records. The putty index record would assist the arrangement of artificial anterior teeth (Figure 20).

**Figure 21:**
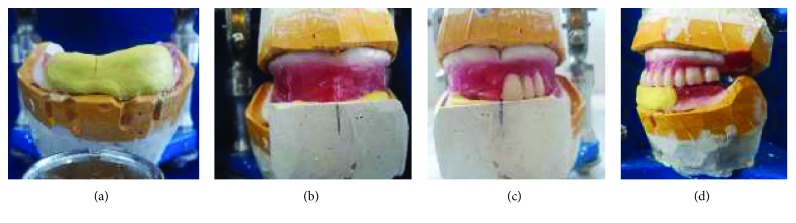
Teeth arrangement. (a) Buccal slots; (b) plaster key; (c) anterior teeth arrangement; (d) upper teeth arrangement.

Before teeth setting, multiple stops were prepared on the exterior surface of the mandibular cast (Figure 21(a)). A buccal key was made with dental stone to mark the buccal inclination of the mandibular incisives and the midline (Figures 21(b) and 21(c)).

**Figure 22:**
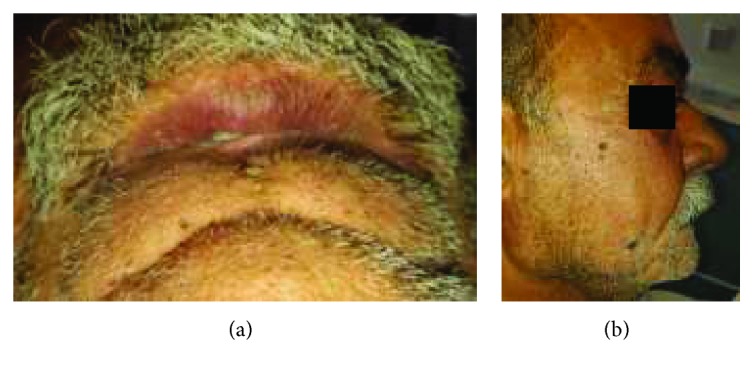
Try-in. (a) Close view of the lips situation; (b) lateral view.

In the same way, the arrangement was completed (Figure 21(d)). Thus, it maintains the anatomic relation between the upper and lower lips in rest and function (Figures 22(a) and 22(b)).

After the try-in stage, complete dentures were fabricated.

## 5. Discussion

Maximal oral openings may range from 36 to 77 mm in healthy persons with mean values from several studies within the 50 to 60 mm “normal range” reported by Posselt. Deviations from normal were classified as either “slight” (Class I, 41 to 50 mm), “moderate” (Class II, 31 to 40 mm), or “severe” (Class III, 30 mm or below) microstomia [10].

During prosthodontic rehabilitation, patients who present with nonideal/pathological conditions for replacement of missing teeth, such as limited mouth opening, should be made aware of the limitations of the prosthesis.

This situation is considered as inconvenient for the patient and challenging for both the dentist and dental technician. Proper treatment planning should be adopted so that a functionally acceptable prosthesis can be fabricated.

So, to plane treatment for a patient with microstomia, it is important to have knowledge about existing complications, alternative methods, and the patient's ability and comfort [11–13].

In some cases, successful prosthetic treatment for patients with microstomia is very difficult without a surgical operation [1].

If not, making complete dentures for them presents difficulties at all stages, from the impressions to the fabrication of the prostheses. It may even be impossible to make it by using conventional methods [2].

Various techniques for making preliminary impressions for patients with microstomia have been presented in the literature. In the treatments described, sectional impression trays and flexible impression trays with semirigid silicone putty impression material were used. A review of the literature shows that different impression methods and designs have been used in a similar procedure. On the one hand, sectional impression trays are advocated. Mirfazaelian, for example, used orthodontic expansion screws to fabricate sectional trays. Cura et al. used metal pins and an acrylic resin block to attach the sections of the impression trays used. Plastic building screws, hinges, and acrylic resin blocks or locking levers had been proposed to attach the sections of the impression trays. On the other hand, Benetti et al. used an entire flexible plastic tray intended for fluoride application to make the preliminary impression [2, 6, 8].

In addition, the fabrication of complete dentures depends on making accurate final impressions that capture the movable tissues in their functional state. For this purpose, a few methods such as flexible and sectional stock trays have been proposed to obtain optimal final impressions [1, 6].

Some amelioration was done to the sectional tray. For example, on one of the sections, Benetti et al. prepared a stepped butt joint to make a definitive impression [8].

An inconvenience of the method of sectional trays is that it was not possible to remove the custom tray in 1 piece; it could be removed as 2 separate segments. This would be done by fracturing the impression material [8].

In addition, it was believed that the cross-section of the sectional impression paste was not wide enough in the midline and that this would negatively affect the stability of the right and the left tray pieces. Also, the two pieces of the tray were arranged in 2 different planes, which affect the proper approximation of two halves of the tray [14].

With patients suffering from microstomia, when the abnormality of the jaw relations is very marked, various modifications in the artificial teeth arrangement may be necessary to obtain satisfactory results in the functional rehabilitation of complete dentures [11].

Because of a lack of denture teeth in appropriate sizes and shapes, conventional denture teeth would be reshaped to appropriate sizes.

In some cases, occlusal areas of posterior artificial teeth were formed with 0-degree cusps to facilitate eccentric mandibular movements [3].

Another way of overcoming the problem in the arrangement of teeth due to the abnormal jaw relation is by using nonanatomic teeth with flate occlusal surfaces. Thanks to this morphology, achieving a satisfactory occlusal relation with the opposing arch will be easier than with semianatomic or anatomic teeth [11].

During the try-in stage, the stability and occlusion of complete dentures would be verified.

Several techniques and designs have been described in the literature for the fabrication of the definitive denture base. The most common procedure used is sectional or foldable denture. During the follow-up period of these types of prostheses, the patient reported dislodgment of the denture during chewing. In fact, Lego pieces for joining the sectional segments do not provide good stability or interlocking, and the result was unsuccessful [5, 12].

Also, earlier reports have fabricated the maxillary complete denture in two parts which creates the problem of midline diastema (esthetics) as well as problems in phonetics [15].

This technique also has some other disadvantages in the term of longer chair time, additional lab time, and material used for the fabrication of sectional denture [16].

Special recommendations must be given to the patient at the prosthesis delivery appointment about how to insert and remove his denture.

1. Involve the patient in the technique: he chooses which denture to insert first. It is preferable to place the maxillary denture prior to inserting the mandibular one and inversely on removing.
2. It is better to repeat inserting and removing prostheses in front of a mirror until he gets acquainted with the new denture manipulation.
3. The denture must be inserted, tilted, and then turned horizontally (90°) to be in place.

## 6. Conclusion

Although patients with microstomia presenting for prosthetic rehabilitation pose a challenge to the clinician at all the clinical procedures, they can conservatively be managed by modifying clinical and laboratory procedures. However, in these modifications, care should be taken to avoid compromising the basic principles of providing optimum function and esthetics to the patient. The authors recommend that selection of an appropriate technique should be based on the case requirement and the operator's skills.
